# The use of automated Ki67 analysis to predict Oncotype DX risk-of-recurrence categories in early-stage breast cancer

**DOI:** 10.1371/journal.pone.0188983

**Published:** 2018-01-05

**Authors:** Satbir Singh Thakur, Haocheng Li, Angela M. Y. Chan, Roxana Tudor, Gilbert Bigras, Don Morris, Emeka K. Enwere, Hua Yang

**Affiliations:** 1 Department of Oncology, University of Calgary, Calgary, Alberta, Canada; 2 Translational Laboratories, Tom Baker Cancer Center, Calgary, Alberta, Canada; 3 Department of Community Health Sciences, University of Calgary, Calgary, Alberta, Canada; 4 Department of Pathology and Laboratory Medicine, University of Alberta, Edmonton, Alberta, Canada; 5 Department of Pathology and Laboratory Medicine, University of Calgary, Calgary, Alberta, Canada; University of South Alabama Mitchell Cancer Institute, UNITED STATES

## Abstract

Ki67 is a commonly used marker of cancer cell proliferation, and has significant prognostic value in breast cancer. In spite of its clinical importance, assessment of Ki67 remains a challenge, as current manual scoring methods have high inter- and intra-user variability. A major reason for this variability is selection bias, in that different observers will score different regions of the same tumor. Here, we developed an automated Ki67 scoring method that eliminates selection bias, by using whole-slide analysis to identify and score the tumor regions with the highest proliferative rates. The Ki67 indices calculated using this method were highly concordant with manual scoring by a pathologist (Pearson’s r = 0.909) and between users (Pearson’s r = 0.984). We assessed the clinical validity of this method by scoring Ki67 from 328 whole-slide sections of resected early-stage, hormone receptor-positive, human epidermal growth factor receptor 2-negative breast cancer. All patients had Oncotype DX testing performed (Genomic Health) and available Recurrence Scores. High Ki67 indices correlated significantly with several clinico-pathological correlates, including higher tumor grade (1 versus 3, P<0.001), higher mitotic score (1 versus 3, P<0.001), and lower Allred scores for estrogen and progesterone receptors (P = 0.002, 0.008). High Ki67 indices were also significantly correlated with higher Oncotype DX risk-of-recurrence group (low versus high, P<0.001). Ki67 index was the major contributor to a machine learning model which, when trained solely on clinico-pathological data and Ki67 scores, identified Oncotype DX high- and low-risk patients with 97% accuracy, 98% sensitivity and 80% specificity. Automated scoring of Ki67 can thus successfully address issues of consistency, reproducibility and accuracy, in a manner that integrates readily into the workflow of a pathology laboratory. Furthermore, automated Ki67 scores contribute significantly to models that predict risk of recurrence in breast cancer.

## Introduction

Breast cancer is the most common form of cancer among women, and is the second-leading cause of cancer-related death worldwide [1]. Treatment decisions for breast cancer are significantly influenced by subtype, which is determined from expression of estrogen receptor (ER), progesterone receptor (PgR), human epidermal growth factor receptor 2 (HER2), and the proliferation marker Ki67 [2]. With advancement of the field of genomics, various genetic tools have been developed which assist in the subtyping of breast cancers, in addition to the immunohistochemical markers listed above. Some of the multigene assays that are currently available for early-stage breast cancers include the Oncotype DX^®^ (Genomic Health Inc.), MammaPrint^®^ (Agendia BV), Prosigna^®^ (PAM50) (NanoString Technologies Inc.) and EndoPredict^®^ (Myriad Genetics Inc.) [3]. The percentage of Ki67-positive tumor cells, or the Ki67 index, is used clinically to distinguish between Luminal A and Luminal B subtypes [4]. Expression of Ki67 is also a good predictor of pathological complete response [5–8], response to chemotherapy [9–14], and likelihood of relapse [15–17].

In spite of the significant body of evidence supporting the clinical application of Ki67, its use is hampered by a few drawbacks. First, there are no universally-accepted guidelines for analysis and clinical interpretation of Ki67 staining. For instance, while the St. Gallen Expert Panel recommended a Ki67 cut-point of 14% for administration of neoadjuvant chemotherapy in 2011 [18], they subsequently withdrew their recommendation of a cut-point, given the variability across different centers [19]. Second, there is no consensus about whether the Ki67 index should be calculated as the percentage of Ki67-positive tumor cells in a tissue section, or from “hot spots” representing tumor regions in which cell proliferation is highest [20, 21]. Third, even with consensus amongst the cut-points and scoring methods, inter-observer variability in manual scoring still exists, even among pathologists [22–25]. While the hot spot method is ideal to represent the highest-proliferating region of a heterogeneous disease, individual biases in selecting these hot spots negatively affect inter-observer concordance in scoring. These issues have prompted the development of several multi-center teams and clinical studies to investigate the utility of automated scoring approaches for Ki67.

In the context of early-stage, ER/PgR-positive breast cancer, adjuvant chemotherapy is only effective in a small subgroup of patients with a high risk of relapse [26–29]. A number of multigene assays have emerged in recent years, which assist in clinical management of early-stage breast cancer patients by estimating the risk of recurrence [30–32]. These assays include Oncotype DX, which is used to support clinical decisions involving the administration of chemotherapy [29, 33–35]. The assay involves the use of gene expression analysis to generate a Recurrence Score; this is a continuous variable, ranging from 0 to 100, that indicates risk of cancer recurrence [29]. Based on the Recurrence Score, patients are classified into low (<18), intermediate (18–30), or high (≥31) risk-of-recurrence groups. The genes assessed by Oncotype DX include those coding for ER, PgR, HER2 and Ki67, with the latter contributing the most to calculation of the Recurrence Score [29, 36, 37]. The high cost of multigene assays (~US$4,000 per patient for Oncotype DX) has prompted multiple groups to create similar risk assessment models, by using biomarker expression data already collected as part of the standard of care [36, 38–41]. Nevertheless, the absence of reliable data on Ki67 remains a weak link with these efforts.

Here, we report the development of a robust automated method of Ki67 scoring, using the HALO^®^ image analysis platform. Our approach combines the biological value of hot spot scoring with whole-slide analysis, to produce results that are unbiased and highly reproducible. We validated the clinical value of Ki67 scores obtained by this approach using standard clinico-pathological data and machine learning algorithms to predict the Oncotype DX risk category of patients in our cohort. These results, upon validation, may provide a method of consistent and accurate Ki67 assessment that could be easily incorporated into current clinical practice.

## Materials and methods

### Patient selection

This study received ethical approval from the Conjoint Health Research Ethics Board at the University of Calgary. Patients included in this retrospective study were 328 women diagnosed with early stage breast cancer (ER/PgR-positive, HER2-negative, lymph node-negative, stages I to II) treated between 2014 and 2016 in Alberta, Canada. Samples from resected tissue specimens were submitted for Oncotype DX testing (Genomic Health). A database (The Alberta Provincial Oncotype DX Database) was developed by HY (Calgary, Canada) and GB (Edmonton, Canada) from the information obtained from the Oncotype DX testing and other clinico-pathological variables (Age, Tumor Size, Tumor Grade, Tumor Nuclear Grade, Tumor Architecture, Tumor Estrogen and Progesterone Receptor levels) available for these patients.

### Specimen selection and immunohistochemistry

For each case of resected breast cancer, the formalin-fixed, paraffin-embedded tissue block with the largest tumor cross-section was identified. One section (4 μm thickness) was stained for hematoxylin & eosin (H&E). A consecutive section was stained with an antibody to Ki67 (clone MIB1, Dako [Santa Clara, California, USA], 1:200 dilution, EnVision FLEX low pH buffer). The slides were counterstained with FLEX hematoxylin (Dako) and permanently mounted per the manufacturer’s protocol.

### Assessment of Ki67 index

Manual scores of Ki67 were generated by a pathologist (HY) from independent counting of at least three high-power fields (40X objective) of the most mitotically active area in a section, with a minimum of 500 invasive tumor cells counted per area. For automated Ki67 assessment, an image analysis algorithm was designed in the HALO^®^ image analysis software platform (version 2.0.1145.14, Indica Labs, Corrales, New Mexico, USA) that identified both Ki67-positive nuclei (brown) and Ki67-negative nuclei (hematoxylin-positive, blue) from slides stained using chromogenic immunohistochemistry. Invasive breast cancers were marked on diagnostic H&E slides by a pathologist (HY). Both the H&E slides and serial tissue sections stained for Ki67 were digitized using an Aperio Scanscope XT slide scanner. The area to be analyzed on the Ki67 image was identified and annotated from the matched H&E using the Image Registration tool in the HALO^®^ software. Once annotated, the entire tumor-bearing region was segmented into 500 × 500 μm square tiles and submitted for image analysis. The results included the number of nuclei detected, as well as the percentage of Ki67-positive nuclei from each tile. The hot-spot Ki67 index for each case was calculated as the mean percentage of Ki67-positive nuclei in the top five tiles having at least 500 cells per tile. The whole-slide Ki67 index was calculated by determining the percentage of Ki67-positive nuclei present in each tile in the marked area, and then calculating the mean per-tile percentage for all tiles on a slide. In this approach, no criterion of having minimum 500 cells per tile was followed, and scores from all tiles in the marked tumor-containing areas were used to calculate the final Ki67 index.

### Statistical analyses

Correlations involving the Ki67 index were statistically tested by Pearson’s correlation coefficient (Pearson’s r and two-tailed P values) and Lin’s concordance correlation coefficient where appropriate using GraphPad PRISM (version 6.0). Correlations between Ki67 indices and various clinico-pathological variables were assessed by nonparametric tests (Kruskal-Wallis to compare among three or more groups; Wilcoxon rank sum for comparison across two groups) for continuous variables and Fisher’s exact test for categorical factors in R (version 3.2.4).

### Random Forest modeling

A Random Forest machine learning paradigm was used to evaluate our ability to predict Oncotype DX risk-of-recurrence categories from available data. All modeling was performed in R (version 3.2.4), using the “randomForest” package (version 4.6–12). The data set was derived from the 199 patients from our cohort for whom we had complete data in all variables. The modelling was designed to predict the Oncotype DX-derived Recurrence Score for all patients in the test group. This was done using, in one instance, the first 13 clinico-pathological variables listed in S2 Table that were obtained from the patients’ charts. The Recurrence Score obtained from this modelling was referred to as the predicted Recurrence Score, pRS. The Oncotype DX reports for each patient included gene expression scores for ER, PgR and HER2, as determined by quantitative RT-PCR [42]. No other Oncotype DX gene expression scores are available in the reports. To determine whether these data would improve the accuracy with which a Random Forest model could predict Recurrence Scores, we created a second modelling instance. In this instance, the contributing variables consisted of the first 13 clinico-pathological variables listed in S2 Table, as well as the Oncotype DX gene expression scores for ER, PgR and HER2, obtained from reports. This Random Forest instance thus consisted of 16 variables that were again used to predict the actual Oncotype DX Recurrence Scores. The predicted Recurrence Scores in this instance were termed pRS_odx_, to differentiate them from the pRS that were derived in the absence of any Oncotype DX data. The predicted Recurrence Scores were then assigned to low, intermediate or high risk-of-recurrence groups, using the standard cut points applied to this test (low <18, intermediate 18–30, high ≥31, range 0–100). Random Forest parameters for *n*_*tree*_, *m*_*try*_ and *sampsize* were optimized using the method of Huang and Boutros [43], and set at *n*_*tree*_ *=* 1000; *m*_*try*_ *=* 15 or 12 for analysis including or not including Oncotype DX ER/PgR/HER2 data, respectively; and *sampsize =* 40. For each round of analysis, data from a randomly-selected 50% of patients were used to train the model, which was then applied to predict Recurrence Scores of the remaining patients. The predicted Recurrence Scores were assigned to risk-of-recurrence groups, as described above. Performance in each round was evaluated by constructing a confusion matrix (2×2 table) that matched each patient’s predicted risk group against the Oncotype DX-determined risk group assignment. For the purposes of assessing predictive performance of the analyses, correct prediction that a patient was of low risk-of-recurrence constituted a true positive test result, and correct prediction that a patient was of high risk constituted a true negative result. Incorrect prediction that a high-risk patient was of low risk constituted a false positive, and incorrect prediction that a low-risk patient was of high risk was a false negative. Patients with intermediate risk (from either actual or predicted Oncotype DX scores) were not included in evaluation of performance. Each round of model-building and prediction also generated data on percentage Increased Mean Squared Error (%IncMSE). This is the difference in mean squared error of the predicted Recurrence Score, between when a specific variable is used in the Random Forest model, and when that variable is randomly permuted [44]. A higher %IncMSE thus indicates greater predictive value of a variable. Results presented are from 1,000 independent rounds of cross-validation. Performance statistics were reported as mean ± standard deviation.

## Results

### Cohort description

The present study involved 328 patients who were treated for hormone receptor-positive, HER2-negative, early-stage breast cancer in Alberta between 2014 and 2016. The clinico-pathological characteristics of these patients are listed in Table 1. All patients in the cohort underwent Oncotype DX genomic testing and had available Recurrence Scores. Using the standard Genomic Health cut-points, there were 185 low-risk (62%), 110 intermediate-risk (28%) and 33 (10%) high-risk patients. Clinico-pathological characteristics of these patients were also grouped by Oncotype DX risk group (S1 Table).

**Table 1:** Clinico-pathological characteristics of patients in the study cohort.

Age at Dx (Years)	Patients	%	n
Mean	55	100	327*
Range	29–85		
**Tumor Size (mm)**			
Mean	18.1	100	316
Range	2.5–57		
**Tumor Grade**			
1	64	20.7	309
2	196	63.4	
3	49	15.8	
**Tumor Nuclear Grade**			
1	32	10.3	309
2	172	55.6	
3	105	33.9	
**Tumor Differentiation**			
1	31	10	309
2	88	28.4	
3	190	61.4	
**Tumor ER Intensity**			
Weak	8	3.2	244
Moderate	13	5.3	
Strong	223	91.3	
**Tumor PgR Intensity**			
Weak	14	6.1	228
Moderate	65	28.5	
Strong	149	65.3	
**Recurrence Score**			
Low (<18)	185	56.4	328
Intermediate (18–30)	110	33.6	
High (>30)	33	10.0	

*: Age at Dx not available for one patient. ER, estrogen receptor; PgR, progesterone receptor

### Performance evaluation of automated Ki67 analysis

**Fig 1:**
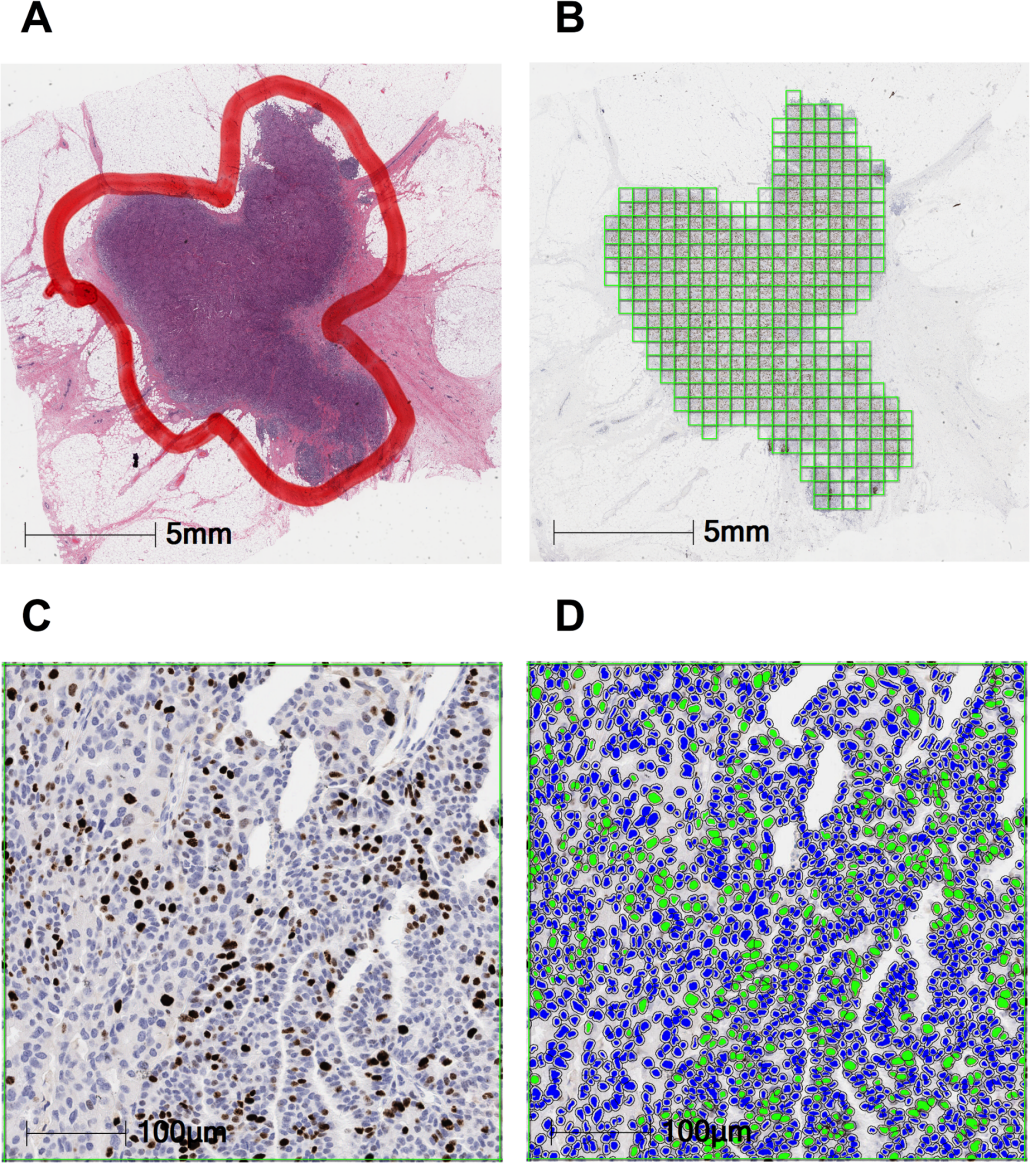
Overview of the automated image analysis workflow. An H&E of the tumor specimen was manually annotated by a pathologist (A). Annotations were transferred to the matching Ki67-stained slide, and segmented into tiles (B). Ki67-positive and -negative nuclei in each tile (C) were identified and counted by the analysis algorithm, which colored them green and blue, respectively (D).

**Fig 2:**
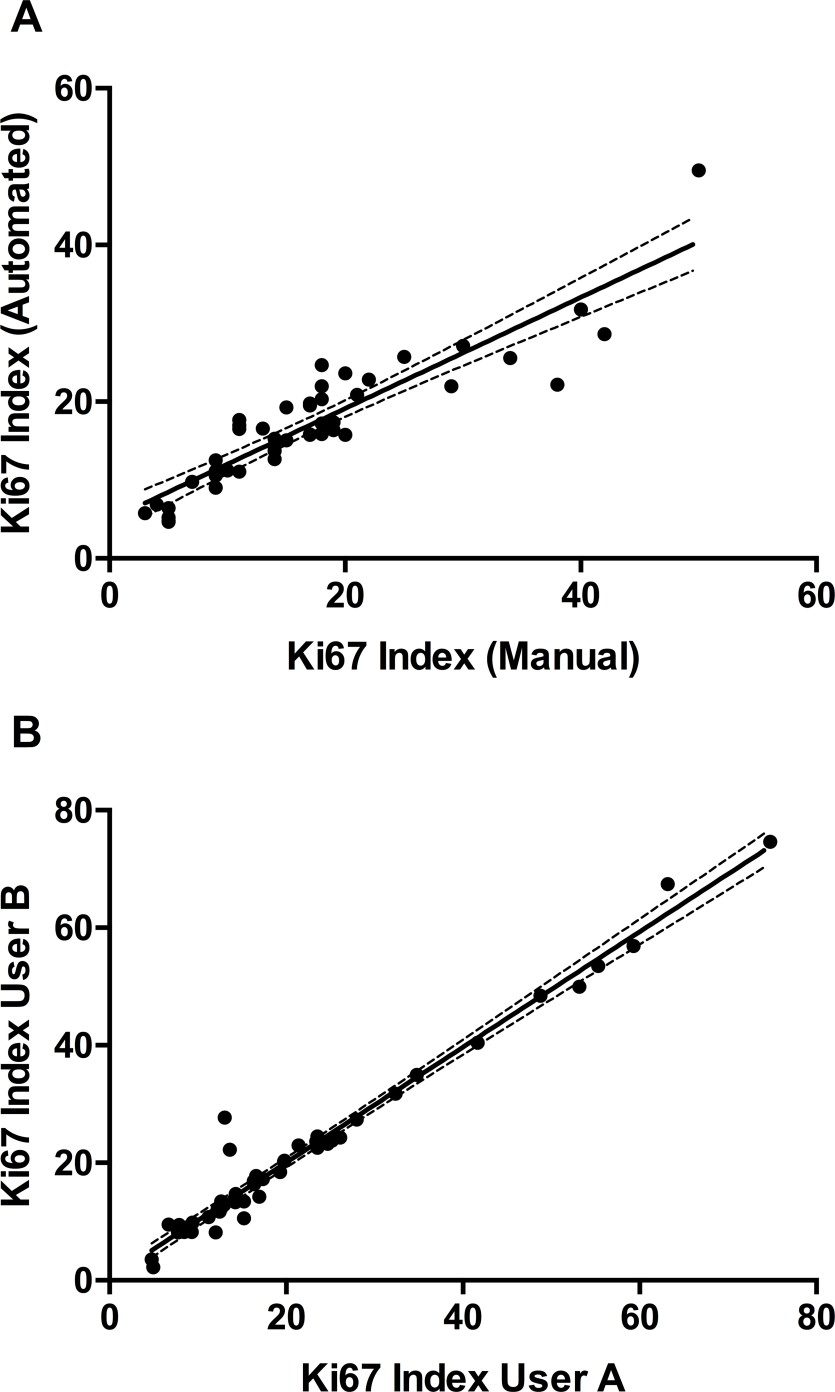
Performance evaluation of automated Ki67 analysis. (A) Comparison of Ki67 indices (n = 45) as assessed by manual and automated scoring. Pearson’s r = 0.909; Lin’s concordance correlation coefficient = 0.881; P<0.001. (B) Assessment of inter-user concordance using the automated analysis (n = 50). Pearson’s r = 0.984; P<0.001. Dashed lines indicate 95% confidence intervals.

An overview of the analysis workflow is shown in Fig 1. Tumor areas were segmented into square tiles as the first step in the automated analysis. The median number of tiles per slide was 328 (range 11–1600, interquartile range 273). Only tiles with ≥ 500 cells were selected for analysis to determine Ki67 index. To evaluate the performance of the automated analysis, we compared the Ki67 indices from 45 randomly selected cases to manual scoring of the same. The manual scoring was performed by a breast pathologist (HY) who was blinded to the automated assay’s results. There was a high correlation between both analysis methods (Fig 2A; Pearson’s r = 0.909, P< 0.001) with high levels of concordance (Lin’s concordance = 0.881).

To test the ease of use and reproducibility of the method developed, 50 cases were selected at random, and subjected to automated analysis separately by one novice and one expert user. As expected, scores from both users were concordant and reproducible with minimal variance (Fig 2B; Pearson’s r = 0.984; P<0.001).

### Clinical validity of whole-slide hot spot analysis

There is no consensus about the relative validity of Ki67 scoring of hot spots, as compared to scoring of the entire slide. The analysis of hot spots allows representation of the most aggressive tumor regions from otherwise heterogeneously-proliferative tumor specimens [20, 45, 46]; however, the utility of this approach is normally marred by inter-user bias. We hypothesized that the elimination of bias, as is the case with our assay, would allow the generation of Ki67 indices that were more strongly correlated with common prognostic factors. Correlations were assessed between whole-slide or hot-spot Ki67 indices and multiple clinico-pathological variables (Table 2). There were interactions between Ki67 indices generated from both approaches (whole-slide and hot-spot) and all three Oncotype DX risk groups. Ki67 indices also correlated positively with the Recurrence Scores, tumor grade and mitotic score, and negatively with ER and PgR Allred scores. There were no notable differences in the extent to which either whole-slide or hot-spot indices interacted with the clinico-pathological variables.

**Table 2:** Assessment of interaction between Ki67 indices and clinico-pathological variables.

RS	Ki67 Index (Whole-slide)	P Value	Ki67 Index (Hot-spot)	P Value
Low	6.86	Int. vs Low = 0.012	15.84	Int. vs Low = 0.005
Intermediate	8.33	High vs Low < 0.001	19.21	High vs Low < 0.001
High	20.21	High vs Int. < 0.001	43.66	High vs Int. < 0.001
**Tumor Grade**				
1	8.15	2 vs 1 = 0.297	16.49	2 vs 1 = 0.704
2	6.53	3 vs 1 < 0.001	16.08	3 vs 1 < 0.001
3	7.46	3 vs 2 < 0.001	33.88	3 vs 2 < 0.001
**ER Score**				
ER<5	16.96	0.004	36.98	0.002
ER = 5	7.05		16.46	
**PgR Score**				
PgR<5	8.98	0.012	20.63	0.008
PgR = 5	6.66		15.27	
**Nuclear Grade**				
1	7.11	2 vs 1 = 0.555	17.10	2 vs 1 = 0.232
2	6.85	3 vs 1 = 0.046	16.06	3 vs 1 = 0.129
3	10.5	3 vs 2 < 0.001	23.83	3 vs 2 < 0.001
**Mitotic Score**				
1	6.63	2 vs 1 < 0.001	15.68	2 vs 1 < 0.001
2	11.95	3 vs 1 < 0.001	25.99	3 vs 1 < 0.001
3	17.54	3 vs 2 = 0.005	35.93	3 vs 2 = 0.008

RS, recurrence score; Int, intermediate-risk group; ER, estrogen receptor; PgR, progesterone receptor.

It is worth mentioning here that Ki67 scores from both approaches (whole-slide and hot-spot) were strongly correlated (Pearson r = 0.938); however, the hot-spot indices were significantly higher than the whole-slide indices across the entire cohort (S1 Fig) and when patients were stratified into Oncotype DX risk-of-recurrence groups (S2 Fig).

**Fig 3:**
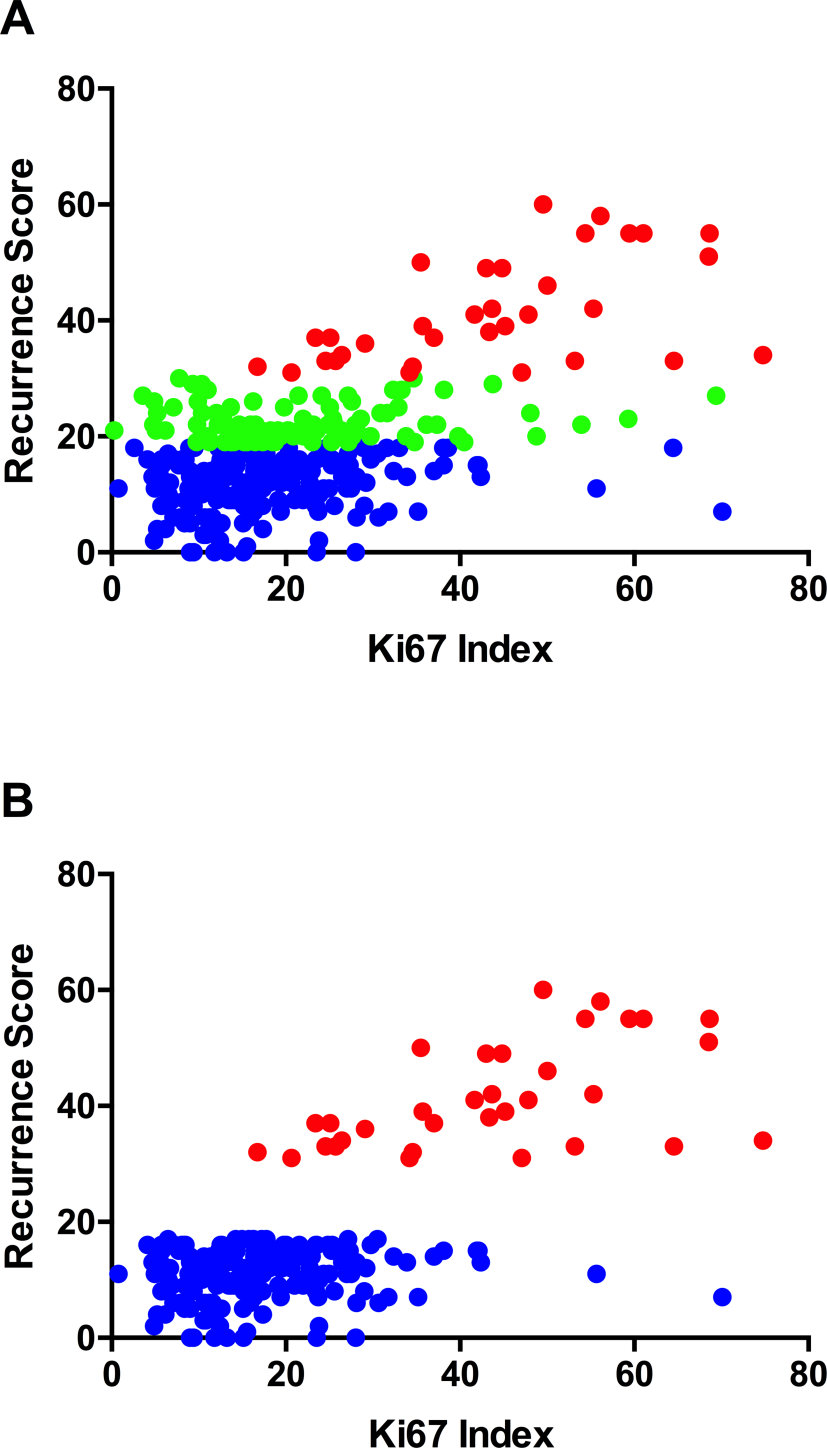
Assessment of correlation between hot-spot Ki67 index and Oncotype DX scores. (A) Plot showing association between Ki67 index and Oncotype DX low-risk (blue), intermediate-risk (green) and high-risk (red) groupings. Pearson’s r = 0.5533; P<0.001. (B) Plot showing association between Ki67 and Oncotype DX low- and high-risk groupings. Pearson’s r = 0.684; P<0.001.

Since Ki67 is the greatest contributor to the Oncotype DX assay’s results, we evaluated the association between hot-spot Ki67 indices and Oncotype DX Recurrence Scores. The correlation between these, while modest, was significant (Fig 3A; Pearson’s r = 0.553, P<0.001). While the distribution of Ki67 indices for low-risk patients overlapped with those for intermediate-risk patients, the median Ki67 indices were significantly higher in the latter (Table 2). In contrast, Ki67 indices of high- and low-risk patients assorted into distinct clusters (Fig 3B). A clear difference in the Ki67 indices between high- and low-risk patients was observed (Pearson’s r = 0.684, P<0.001), with 97% patients in the high-risk category having Ki67 indices over 20%.

### The use of machine learning to infer Oncotype DX risk groups

**Fig 4:**
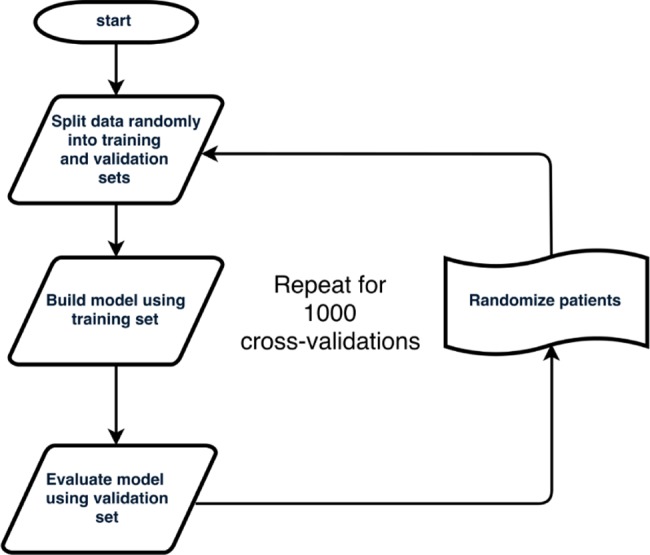
Outline of Random Forest training and evaluation workflow.

**Fig 5:**
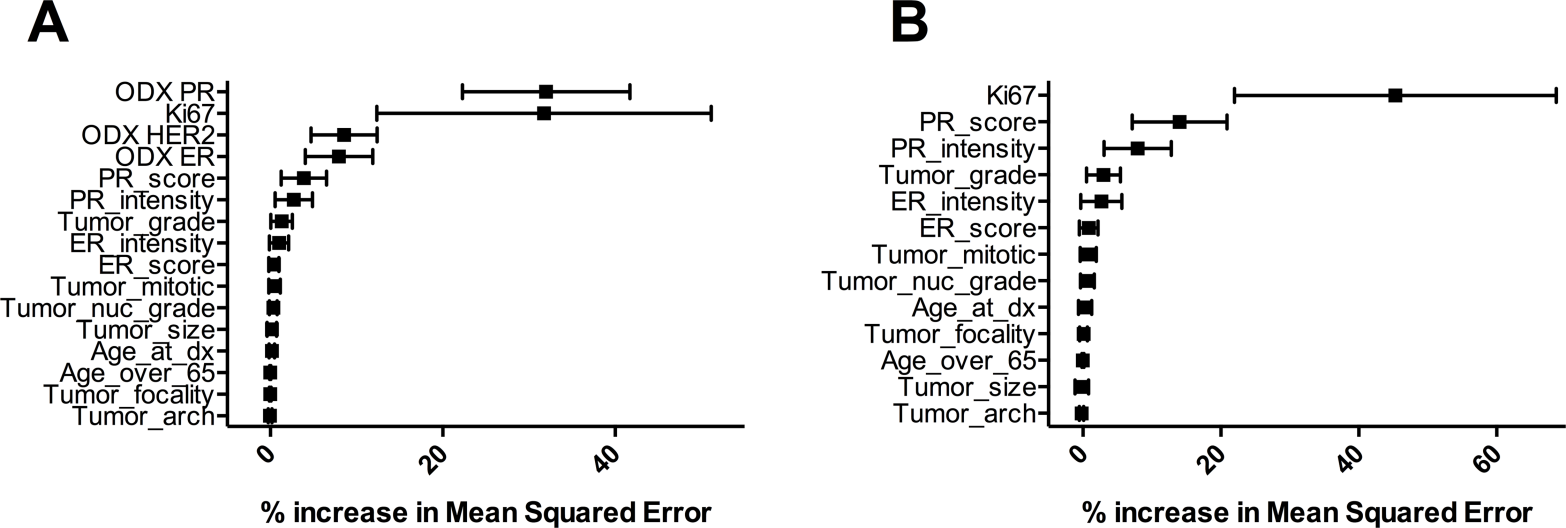
Contribution of individual variables to the accuracy of the respective Random Forest models, as assessed by increases in mean squared error for models created without each variable. Graphs represent models after 1,000 cycles of validation trained with both clinico-pathological data and Oncotype DX expression data for ER, PgR and HER2 (A) or with clinico-pathological data alone (B). Error bars represent standard deviation from the mean. ER intensity, estrogen receptor staining intensity; ER score, estrogen receptor expression score (immunohistochemistry); PR intensity, progesterone receptor staining intensity; PR score, progesterone receptor expression score (immunohistochemistry); ODX ER, Oncotype DX estrogen receptor gene expression score; ODX HER2, Oncotype DX HER2 expression score; ODX PR, Oncotype DX progesterone receptor gene expression score; Tumor_arch, tumor differentiation score; Tumor_nuc_grade, tumor nuclear grade.

Ki67 is the main contributor to the Oncotype DX recurrence score [29, 36, 37], and markers of proliferation similarly contribute to the risk-of-recurrence assessments of other multigene assays [30]. We asked if an integrated machine learning analysis of clinico-pathological data, including the Ki67 scores generated by our automated assay, could provide useful information about recurrence risk in these patients. We created and evaluated a Random Forest machine learning model, using a subset of 199 patients for whom we had complete data sets. While we used all available Oncotype DX data for model training, we focused our assessment of model performance on high- and low-risk patients. This is because the intermediate risk patients constitute an ambiguous group from both predictive and treatment standpoints [36, 41]. The outline for model training, evaluation and cross-validation is shown in Fig 4, the variables used are listed in S2 Table, and the summary results from 1,000 rounds of cross-validation are in Table 3. Predicted Recurrence Scores were obtained using 13 clinico-pathological variables alone (pRS) or in addition to the gene expression scores for ER, PgR and HER2 as obtained from the Oncotype DX reports (pRS_odx_). Random Forest models trained with Oncotype DX-derived expression data for ER, PgR and HER2 performed better than models trained without these data; however, the differences were modest, particularly for accuracy (1.0%) and negative predictive value (6.3%). Similar results were obtained when Ki67 indices from whole-slide analyses were used in place of the hot-spot data (Table 3). To determine which variables contributed the most to the accuracy of the model, we calculated the increase in mean squared error, which indicates the degree to which the model’s accuracy would decrease if a specific variable were omitted. In the pRS_odx_ models, PgR (determined by RT-PCR in the Oncotype DX assay) and Ki67 were the greatest contributors, together accounting for approximately 70% of the model’s accuracy (Fig 5A). In the absence of Oncotype DX data, Ki67 was the highest contributor towards model accuracy, with its loss creating a mean squared error increase of 45.3% (Fig 5B).

**Table 3:** Summary performance of the Random Forest models predicting Oncotype DX risk groups. Recurrence Scores were predicted using the clinico-pathological variables listed in S2 Table alone (pRS), or using the S2 Table variables in addition to gene expression scores for ER, PgR and HER2 that were included in the official Oncotype DX reports (pRS_odx_). Evaluation of performance of the Random Forest models was based on the extent to which the models correctly predicted, or failed to predict, each patient’s actual low- or high-risk Oncotype DX category. Values represent the mean outcomes ± standard deviations over 1,000 testing iterations.

	Using hot-spot Ki67 scores		Using whole-slide Ki67 scores	
Parameter	pRS_odx_ vs. RS (%)	pRS vs. RS (%)	pRS_odx_ vs. RS (%)	pRS vs. RS (%)
**Accuracy**	97.8 ± 1	96.8 ± 2	98.2 ± 2	97.2 ± 2
**Sensitivity**	99.4 ± 0.9	98.7 ± 1	99.9 ± 0.5	99.5 ± 1
**Specificity**	81.9 ± 16	80.1 ± 17	80.2 ± 17	75.5 ± 19
**Positive predictive value**	98.2 ± 1	97.8 ± 1	98.2 ± 2	97.5 ± 2
**Negative predictive value**	94.1 ± 10	87.8 ± 12	98.8 ± 6	94.4 ± 12

RS, Oncotype DX Recurrence Score.

## Discussion

Ki67 is commonly used as a marker for proliferation, and has significant value as a prognostic biomarker in breast cancer [10, 20, 23, 47]. Nevertheless, it has proven challenging to establish standards for the quantification and interpretation of Ki67 in clinical practice. In routine diagnostic procedures, Ki67 scoring, as performed manually by a pathologist, involves visual inspection of a limited number of tumor cells [23]. This method suffers from considerable inter-user discordance, creating difficulties in identification and validation of cut-points. In this report, we present a simple, automated scoring method for Ki67 assessment which addresses some of the key challenges currently associated with the analysis of this biomarker. Furthermore, this method combines the advantages of an unbiased whole-slide analysis with the clinical value of identifying the hot spots of highest tumor proliferation. The method features very high concordance against expert manual scoring, and between users. Finally, the Ki67 indices derived from this method correlate as expected with other clinico-pathological variables, and allow accurate inference of Oncotype DX risk-of-recurrence groups.

While several other groups have applied automated methods to the quantification of Ki67 [23, 48, 49], including some large multi-center studies [50, 51], our approach addresses a number of limitations evident in those studies. Many hormone receptor-positive breast cancers exhibit differences in proliferative rate across their spatial extents. In such tumors, the hot spots of higher-than-average proliferative indices are clinically meaningful [52, 53]. Consequently, automated analysis of tissue microarrays, or of manually-selected tumor regions, incur the same potential for selection bias as do manual scoring approaches [20, 54]. We addressed this problem by segmenting each slide image into tiles prior to analysis; since the entire image was subsequently analyzed, the hot spots emerged naturally as the tiles with the highest Ki67 scores. Furthermore, as the Ki67 index represents the mean of the top five tiles, we captured Ki67 data from multiple hot spots across each slide, as well as from non-hot spot regions. The result was a Ki67 index that accounted for proliferative heterogeneity without sacrificing either accuracy or robustness of performance. In contrast, for whole-slide Ki67 index, Ki67 scores from the tiles comprising the entire marked area on the slide were assessed.

The key advantage of computer-assisted analysis of Ki67 is consistency of scoring, particularly between users [23, 25]. The version of the analysis algorithm described here represented the end-point of an optimization process performed on a subset of slides, which was used to analyze all 328 patient samples without any extra optimization. While the automated analysis demonstrated high concordance against a pathologist’s manual scoring, the key demonstration of utility was in the inter-user comparison. With less than 15 minutes of educational-instruction, a novice user of the software was successfully trained to perform an analysis of 50 slides, obtaining almost perfect concordance with an expert user. The only manual intervention required, and thus the only sources of inter-user variance, were in transferring the pathologist’s annotations to the digital images, and visual quality assessment of the top-scoring tiles. The automated analysis thus demonstrates a number of features–validity against the gold standard, reproducibility, and ease of use–that could facilitate its implementation in a clinical environment.

While other studies have demonstrated the prognostic value of Ki67 as assessed by whole slide- and hot spot-focused analysis methods, the value of the hybrid analysis approach we applied in this study was not immediately apparent. We consequently used multiple methods to evaluate the validity of our Ki67 data. Previous studies highlighted associations between tumor proliferation and a variety of clinico-pathological endpoints, such as tumor grade, stage, and ER/PgR expression [55–57]. We observed in this study that Ki67 indices, derived from either whole-slide or hot-spot analysis approaches, correlated significantly with all of these. It is thus likely that, at least for the purposes highlighted in this study, whole-slide and hot-spot Ki67 analyses may be equally useful, so long as they are accurately determined. Ki67 is the greatest contributor to Oncotype DX recurrence score [31], and a strong correlation between Ki67 index and recurrence score could provide evidence supporting the clinical validity of our approach [37, 48, 58]. We observed a particularly strong correlation between Ki67 index and the high and low Oncotype DX risk-of-recurrence categories. Finally, we applied a multivariate machine learning approach to determine if Ki67 indices could contribute to prediction of these Oncotype DX categories. Remarkably, a model trained solely on clinico-pathological data, biomarker expression and Ki67 indices in both hot spot and whole-slide approaches predicted high- and low-risk of recurrence groups with 97% accuracy. In this context, Ki67 index was the most significant contributor to the accuracy of the model. Our results are similar to those published recently by Kim and colleagues [41], who used an identical machine learning approach to predict Oncotype DX risk-of-recurrence status from clinico-pathological and biomarker expression data. Given that Oncotype DX prediction methods, including the linear regression Magee equations [36, 40, 59], utilize potentially unreliable data from manual Ki67 scoring, it is likely that the accuracy of such methods would be significantly improved with robust Ki67 data from automated analyses.

Our study has a number of shortcomings that need to be addressed in the future. First, this study was conducted in a single academic center; consequently, it is not clear what logistical problems may occur in implementing this method in a multi-institutional setup. Secondly, the manual scores in this study were generated by a single pathologist so the exact measure of the inter-observer variability in calculating Ki67 index manually cannot be obtained and compared with the automatically generated Ki67 indices. Also, the manual scores were generated using a single tissue section from each patient with the largest tumor cross sectional area. It is possible that the single section selected may not represent the true mitotic nature of the tumor, due to intra-tumoral heterogeneity. The methodology as presented involves the use of expensive, proprietary software (HALO^®^). However, the fundamentals should be reproducible on a sufficiently robust open-source framework, such as CognitionMaster [41, 60]. This needs to be tested and could determine the extent of variability in results amongst different image analysis software. Furthermore, development and assessment of the machine learning model was only possible on a subset of patients (199) for whom we had complete data sets, since the modeling paradigm is not tolerant of missing data. In this, as in similar studies [36, 40, 59], there were few patients in the high risk-of-recurrence group; further testing of the model needs to be carried out in a larger patient population, with a more equitable distribution of patients in all three risk categories. The small cohort size also precluded our ability to evaluate a locked-down Random Forest model on a separate, independent group of patients. Such an approach would constitute a more thorough validation of the concepts introduced in the current proof-of-principle work.

In the present study, we have addressed the issue of Ki67 scoring which to date remains a major hurdle in clinical use of Ki67 as a biomarker. We present an automated, robust and reproducible method for quantification of Ki67 from whole-slide sections of breast cancer. The consistency and ease-of-use of this Ki67 analysis approach may facilitate the standardized scoring of this biomarker across multiple clinical centers. We show that the Ki67 index derived with this method contributes significantly to prediction of Oncotype DX risk-of-recurrence status, when implemented in a multivariate machine learning model. The utility of predictive modeling, bolstered by the availability of accurate Ki67 data, may reduce the need for expensive multigene assays to assess risk of recurrence.

## Supporting information

S1 Fig Comparison of Ki67 indices from whole-slide and hot-spot analyses. (DOCX)

S2 Fig Comparison of mean Ki67 indices from whole-slide and hot-spot analyses, across different Oncotype DX risk-of-recurrence groups. Error bars represent standard error of mean; * = P<0.05.
(DOCX)

S1 Table Clinico-pathological characteristics of patients, grouped by Oncotype DX risk-of-recurrence group. P values were generated by Kruskal-Wallis, Wilcoxon or Fisher’s exact tests as appropriate.
(DOCX)

S2 Table Variables used as model inputs in the Random Forest analyses. Oncotype DX Recurrence Score was the predicted variable in all cases.
(DOCX)
