# Supplementary figures and images for: The use of automated Ki67 analysis to predict Oncotype DX risk-of-recurrence categories in early-stage breast cancer
Source: PLoS One. 2018 Jan 5;13(1):e0188983. doi: 10.1371/journal.pone.0188983 (PMC5755729; doi:10.1371/journal.pone.0188983)

**S1 Figure. Comparison of Ki67 indices from whole-slide and hot-spot analyses.**


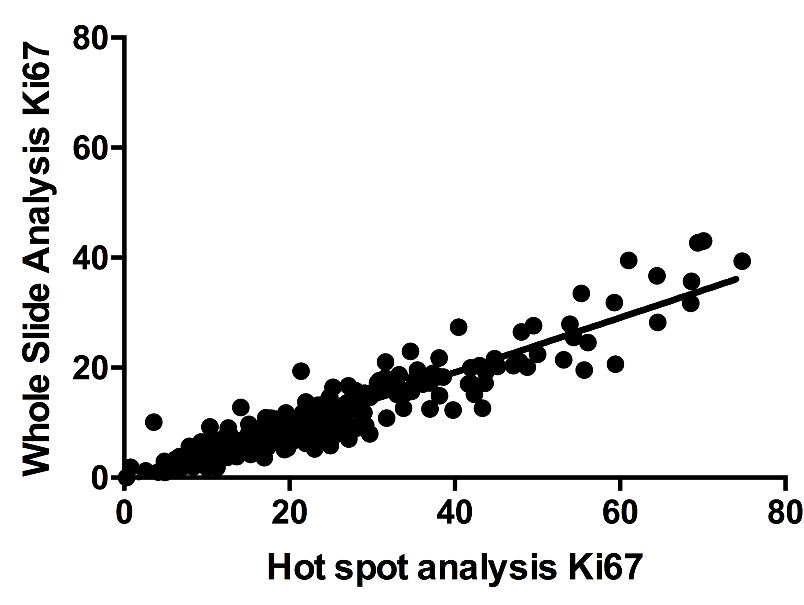


**Pearson’s r = 0.938**

Supplement: S1 Fig — (DOCX) [file pone.0188983.s001.docx]
