# Supplementary material for: The use of automated Ki67 analysis to predict Oncotype DX risk-of-recurrence categories in early-stage breast cancer
Source: PLoS One. 2018 Jan 5;13(1):e0188983. doi: 10.1371/journal.pone.0188983 (PMC5755729; doi:10.1371/journal.pone.0188983)
Supplement: S2 Fig — Error bars represent standard error of mean; * = P<0.05. (DOCX) [file pone.0188983.s002.docx]

**S2 Figure. Comparison of mean Ki67 indices from whole-slide and hot-spot analyses, across different Oncotype DX risk-of-recurrence groups.** Error bars represent standard error of mean; * = P<0.05

**
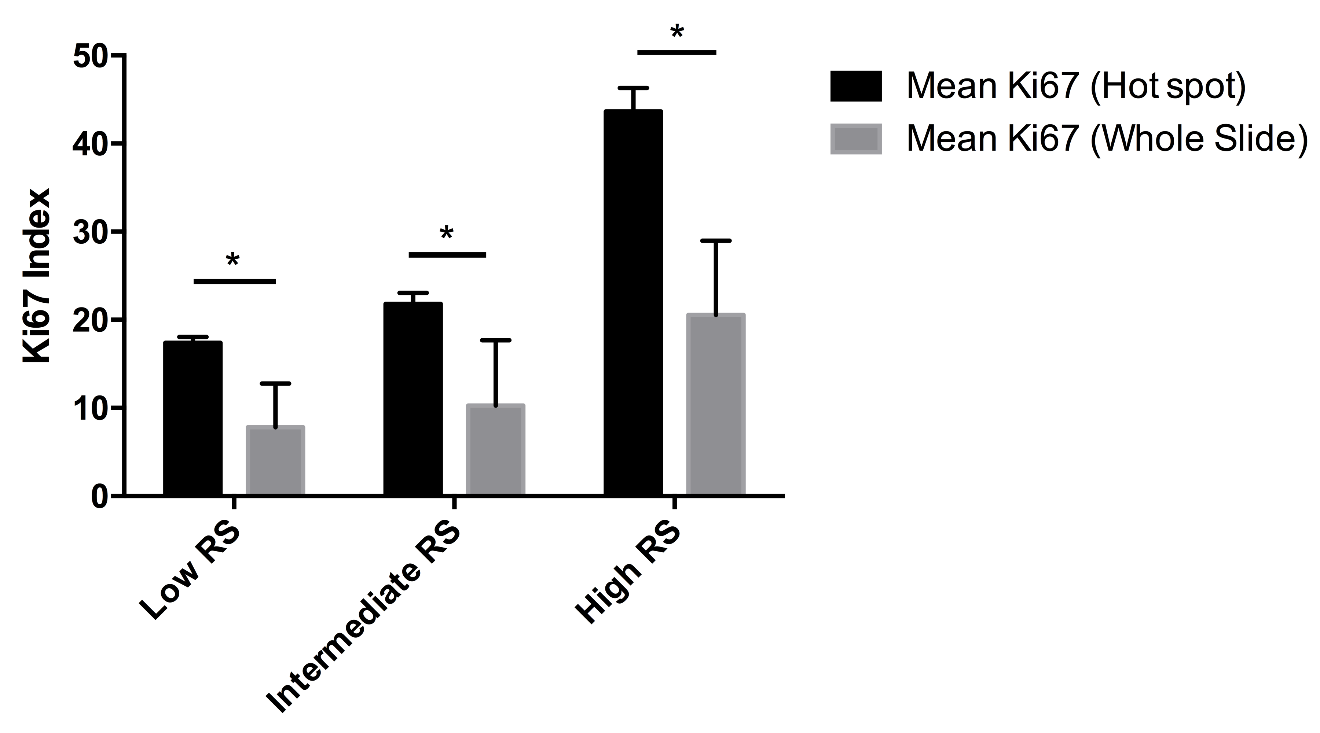
**
