# Supplementary material for: The use of automated Ki67 analysis to predict Oncotype DX risk-of-recurrence categories in early-stage breast cancer
Source: PLoS One. 2018 Jan 5;13(1):e0188983. doi: 10.1371/journal.pone.0188983 (PMC5755729; doi:10.1371/journal.pone.0188983)
Supplement: S1 Table — P values were generated by Kruskal-Wallis, Wilcoxon or Fisher’s exact tests as appropriate. (DOCX) [file pone.0188983.s003.docx]

**S1 Table. Clinico-pathological characteristics of patients, grouped by Oncotype DX risk-of-recurrence group.** P values were generated by Kruskal-Wallis, Wilcoxon or Fisher’s exact tests as appropriate.

| **Recurrence Score** | **RS<18** | **RS 18-30** | **RS>30** | **P value** |
| --- | --- | --- | --- | --- |
| Low | 185 | 0 | 0 | - |
| Intermediate | 0 | 110 | 0 |  |
| High | 0 | 0 | 33 |  |
| **Age (Years)** |  |  |  | **P value** |
| Average | 57.34 | 56.96 | 59.18 | 0.449 |
| Missing | 1 | 0 | 0 |  |
| **Age at Dx (Years)** |  |  |  | **P value** |
| Average | 55 | 54 | 56 | 0.341 |
| Missing | 1 | 0 | 0 |  |
| **Tumor Grade** |  |  |  | **P value** |
| 1 | 43 | 16 | 5 | <0.001 |
| 2 | 119 | 64 | 13 |  |
| 3 | 10 | 26 | 13 |  |
| Missing | 13 | 4 | 2 |  |
| **Tumor Nuclear Grade** |  |  |  | **P value** |
| 1 | 20 | 8 | 4 | <0.001 |
| 2 | 111 | 53 | 8 |  |
| 3 | 41 | 45 | 19 |  |
| Missing | 13 | 4 | 2 |  |
| **Tumor Mitotic Score** |  |  |  | **P value** |
| 1 | 142 | 66 | 11 | <0.001 |
| 2 | 22 | 32 | 10 |  |
| 3 | 8 | 8 | 10 |  |
| Missing | 13 | 4 | 2 |  |
| **Tumor Differentiation** |  |  |  | **P value** |
| 1 | 21 | 7 | 3 | 0.259 |
| 2 | 53 | 25 | 10 |  |
| 3 | 98 | 74 | 18 |  |
| Missing | 13 | 4 | 2 |  |
| **Tumor ER intensity** | **RS<18** | **RS 18-30** | **RS>30** | **P value** |
| Weak | 0 | 3 | 5 | <0.001 |
| Moderate | 7 | 3 | 3 |  |
| Strong | 134 | 74 | 15 |  |
| Missing | 44 | 30 | 10 |  |
| **Tumor PR intensity** |  |  |  | **P value** |
| Weak | 4 | 7 | 3 | <0.001 |
| Moderate | 30 | 27 | 8 |  |
| Strong | 105 | 40 | 4 |  |
| Missing | 46 | 36 | 18 |  |
| **ER Score (Allred)** |  |  |  | **P value** |
| <5 | 2 | 7 | 8 | <0.001 |
| =5 | 139 | 73 | 17 |  |
| Missing | 44 | 30 | 8 |  |
| **PR Score (Allred)** |  |  |  | **P value** |
| <5 | 32 | 40 | 21 | <0.001 |
| =5 | 107 | 36 | 3 |  |
| Missing | 45 | 31 | 9 |  |
| **Oncotype DX ER score** |  |  |  | **P value** |
| Average | 10.3 | 9.57 | 8.65 | 0 |
| Missing | 14 | 7 | 4 |  |
| **Oncotype DX PgR score** |  |  |  | **P value** |
| Average | 8.16 | 6.73 | 4.77 | 0 |
| Missing | 14 | 7 | 4 |  |
| **Oncotype DX HER2 score** |  |  |  | **P value** |
| Average | 9.11 | 8.92 | 8.6 | 0.001 |
| Missing | 14 | 7 | 4 |  |
| **Tumor Focality** |  |  |  | **P value** |
| Single | 139 | 91 | 25 | 0.073 |
| Multifocal | 42 | 13 | 7 |  |
| Missing | 4 | 6 | 1 |  |
| **Tumor Size (Avg mm)** |  |  |  | **P value** |
| Average | 18.42 | 17.43 | 18.35 | 0.479 |
| Missing | 6 | 5 | 1 |  |
