# Supplementary material for: The use of automated Ki67 analysis to predict Oncotype DX risk-of-recurrence categories in early-stage breast cancer
Source: PLoS One. 2018 Jan 5;13(1):e0188983. doi: 10.1371/journal.pone.0188983 (PMC5755729; doi:10.1371/journal.pone.0188983)
Supplement: S2 Table — Oncotype DX Recurrence Score was the predicted variable in all cases. (DOCX) [file pone.0188983.s004.docx]

**S2 Table. Variables used as model inputs in the Random Forest analyses.** Oncotype DX Recurrence Score was the predicted variable in all cases.

| **Variable** | **Variable type/options** | **Used in prediction of** |
| --- | --- | --- |
| Age at Dx | Continuous | pRS and pRS_odx_ |
| Age over 65 | Categorical (yes/no) | pRS and pRS_odx_ |
| Tumor size | Continuous | pRS and pRS_odx_ |
| Tumor grade | Categorical (1/2/3) | pRS and pRS_odx_ |
| Tumor mitotic grade | Categorical (1/2/3) | pRS and pRS_odx_ |
| Tumor nuclear grade | Categorical (1/2/3) | pRS and pRS_odx_ |
| Tumor differentiation | Categorical (1/2/3) | pRS and pRS_odx_ |
| Tumor focality | Categorical  (single/multifocal) | pRS and pRS_odx_ |
| ER score | Continuous | pRS and pRS_odx_ |
| ER intensity | Categorical  (strong/moderate/weak) | pRS and pRS_odx_ |
| PgR score | Continuous | pRS and pRS_odx_ |
| PgR intensity | Categorical  (strong/moderate/weak) | pRS and pRS_odx_ |
| Ki67 score | Continuous | pRS and pRS_odx_ |
| Oncotype DX ER | Continuous (range 3.7 – 12.5) | pRS_odx_ |
| Oncotype DX PgR | Continuous (range 3.2 – 10.0) | pRS_odx_ |
| Oncotype DX HER2 | Continuous (range 7.6 – 13.0) | pRS_odx_ |
